# Exploring human-animal interactions beyond academic research: A rapid review of grey literature

**DOI:** 10.1017/awf.2025.10029

**Published:** 2025-07-28

**Authors:** Siyu Ru, Daniel Villarreal Hernandez, Szymon Parzniewski, Haorui Wu

**Affiliations:** 1 School of Social Work, Dalhousie University, Halifax, NS B3H 4R2, Canada; 2 Bedford Parks Animal Hospital, Bedford, NS B4B 2E6, Canada

**Keywords:** Animal welfare, grey literature review, human-animal interactions, human-animal welfare, public education, resilience

## Abstract

Increasing recognition of the diverse benefits of human-animal interactions (HAIs) has propelled related studies. Although most of the benefits have been illustrated by academic literature (e.g. journal articles, academic theses, and project reports), the grey literature contributes to a comprehensive understanding of HAIs and offers new perspectives, informing prospective research, practices, and policies. Adapting the Systematic Reviews and Meta-Analyses (PRISMA) approach, this rapid review examined 151 articles covering HAIs from 2016–2022. The univariate analysis results revealed that the sources covered various animal species (e.g. dogs, cats, birds), types of animals (e.g. companion animals, therapy animals, zoo animals), and vulnerable populations (e.g. older adults, people with disabilities). HAIs could be found across different settings, such as households, schools, healthcare facilities, and more. The thematic analysis identified three primary categories associated with HAIs’ benefits in public education: (1) HAIs benefit the well-being of individuals, families, and animals; (2) HAIs promote prosocial behaviours and community development; and (3) HAIs improve disaster preparedness and response. The results highlight the multifaceted positive influences of HAIs on human well-being, animal welfare, and building healthy and resilient communities. Grey literature plays an essential role in knowledge mobilisation through public education, promoting the interconnectedness between human well-being and animal welfare.

## Introduction

Human-animal interactions (HAIs) refer to a wide range of activities and relationships between human and animals (AVMA undated), such as companion animal guardianship, farm animals, agricultural animal research, and wildlife conservation efforts (Hosey & Melfi 2014). HAIs can “*be positive, negative, or neutral for either party*” and “*occur in individual, community, or societal contexts*” (AVMA undated). The increasing attention to HAIs in academic research has given rise to the field of human-animal studies, which explores HAIs through an interdisciplinary approach (Irvine 2008). HAIs, particularly living and interacting with companion animals, have proven in many instances to positively influence individual well-being as well as community integration by providing physical health benefits, mental health benefits, and social benefits (Carr *et al.* 2018; Fine 2019; Wu *et al.* 2023a). For instance, research has demonstrated that interacting with companion animals can be a protective factor against cardiovascular diseases (Hodgson *et al.* 2015). Compared with people living without companion animals, companion animal guardians (CAGs), especially dog guardians, have a higher level of physical activity through activities, such as walking, playing with, and caring for their animals (Rijken & Beek 2011). HAIs also contribute to lower heart rates and blood pressure, as well as a decreased frequency of doctor visits among CAGs (Zheng *et al.* 2005). Companion animals offer unconditional love and constant companionship, which enhance the mental well-being of their guardians (Carr *et al.* 2018; Fine 2019). Research has shown that CAGs recover faster from stressful events than people without companion animals, suggesting HAIs help build people’s resilience capacity (Rijken & Beek 2011; Irvine & Cilia 2017). Taking care of the animal fosters a sense of purpose and fulfillment, contributing to a higher level of self-worth and self-esteem (Carr *et al.* 2018; Gibson *et al.* 2022). Companion animals act as a “social lubricant” and “social facilitator”, often generating a conversation and attracting positive attention (Wood *et al.* 2005). The social benefits of HAIs lead to increased social interactions and contacts, which help people build new friendships and expand their social networks (Hodgson *et al.* 2015). By bringing people together in shared spaces, such as parks, neighbourhoods, and community events, companion animals contribute to a greater sense of belonging and stronger community integration and cohesion (Wood *et al.* 2005).

The positive influences of HAIs have also been adopted in practices, namely, animal-assisted therapy and animal-assisted interventions. Researchers and practitioners have utilised such therapy and interventions as an approach for treating substance use disorders (Trujillo *et al.* 2020), as a harm reduction strategy in the prison system to improve prisoner-staff relations and in addressing mental health issues related to imprisonment (Gibson *et al.* 2023), as well as helping enable veterans to cope with post-traumatic stress disorder (PTSD) (Farmer 2021) and supporting children with autism (Anderson & Meints 2016). These applications highlight the therapeutic potential of HAIs across diverse populations and settings.

While HAIs offer numerous benefits, they also present significant challenges (Blazina 2019). One major challenge faced by companion animal guardians (CAGs) is the loss of companion animals and subsequent bereavement, which has received more attention in academia in recent years (Gibson *et al.* 2022). The loss of companion animals can lead to intense grief, distress, and even PTSD (Adrian *et al.* 2009). If the deep bond between CAGs and their animals is not recognised and respected, CAGs may experience disenfranchised grief, grief that is not openly acknowledged, which can further compromise their health and well-being (Adrian *et al.* 2009; Gibson *et al.* 2022). Additionally, research has identified ‘the link’ between animal abuse and violence against humans. Cruelty to animals co-exists with other forms of violence, such as child abuse, domestic violence, and elder abuse (Irvine 2008). Flynn (2000) interviewed women suffering from intimate partner violence and found that their partners harmed the companion animals to threaten and emotionally abuse the participants. In many cases, companion animals provided emotional support to these battered women and impacted their decisions to seek help or go to shelters (Flynn 2000).

Another concern about HAIs is animal suffering and exploitation. Drawing on intersectional theory, researchers argue that speciesism — a type of discrimination based on species (Nibert 2002) — is another form of oppression, paralleling racism, sexism, classism, ablism, and ageism (Irvine 2008). Therefore, promoting animal welfare, typically used in ethical, scientific, and policy discussions to refer to animals’ physical and psychological states, requires addressing the unbalanced power relationship between animals and humans (Nibert 2002). N.B. We acknowledge the asymmetry in the use of the terms ‘welfare’ for animals and ‘well-being’ for humans; however, this terminology reflects established conventions in both academic and practice-based literature. The use of “animal welfare” and “human well-being” aligns with how these concepts are most commonly framed and understood in the reviewed sources, ensuring consistency and relevance to the language employed across the grey literature. The One Welfare framework suggests the interconnectedness between animal welfare, human well-being, and the environment (Mackenzie & Jeggo 2019). Thus, well-being is used in this manuscript to refer more broadly to human experience, encompassing emotional, psychological, and social dimensions. Protecting animal welfare will ultimately contribute to enhanced human well-being and community health. Given this interdependence, knowledge mobilisation based on current research findings is crucial to promoting public education and increasing public awareness of human-animal welfare. Grey literature, referring to materials produced outside of traditional academic peer-review processes, such as reports, magazine articles, newsletters, etc (Adams *et al.* 2017), may help disseminate current knowledge and highlight emerging trends, making the information more accessible for practitioners, policy-makers, and the general public. Grey literature has typically not been subject to the same rigorous evaluation process as academic journal articles. As a result, while it can offer valuable insights — especially on emerging practices, community experiences, and real-world applications — the conclusions drawn from grey literature should be interpreted with caution. Confidence in these findings depends on factors such as the credibility of the source, transparency of methods, and alignment with peer-reviewed evidence. Therefore, grey literature can complement academic research, but should not be solely relied upon for drawing generalisable or definitive conclusions.

Literature review papers have been done to synthesise the existing knowledge of HAIs from academic literature, covering topics such as trends and progress of HAIs research (Griffin *et al.* 2019), the impacts of HAIs on human-animal welfare (Zulkifli 2013), the benefits and challenges of HAIs in disaster settings (Wu *et al.* 2023b), and the efficacy of animal-assisted therapy and animal-assisted interventions (Bert *et al.* 2016). However, reviews of grey literature on HAIs remain scarce. This article conducts a rapid review to examine recent grey literature and explore its role in promoting human-animal welfare. Specifically, this rapid review article aims to: (1) review the publicly available grey literature on HAIs; (2) summarise the focus of the grey literature by identifying key themes; and (3) investigate grey literature’s role in public education and provide recommendations for future research and practices regarding HAIs.

## Materials and methods

We adapted the Preferred Reporting Items for Systematic Reviews and Meta-analyses (PRISMA) approach (Page *et al.* 2021) for this rapid review of grey literature and followed three steps of the PRISMA guidelines to search, screen, and analyse recent grey literature.

### Step 1: Data searching

We searched three databases — Eureka, Factiva, and NexisUni — to identify grey literature. The search functionalities varied slightly across the Eureka, Factiva, and NexisUni databases, requiring adjustments to ensure consistency and relevance in the results retrieved. One key difference lies in how each platform handles hyphenation: for example, “human-animal” may be treated as a distinct term in one database but separated into “human” and “animal” in another, potentially affecting the specificity of the search. Quotation marks also function differently — while they are generally used to search for exact phrases, some databases (such as Factiva) may treat quoted terms more strictly, whereas others (like Eureka) might apply broader proximity logic. Lastly, truncation symbols (e.g. the asterisk *) are not uniformly recognised or may operate with different rules across platforms. For instance, NexisUni supports basic truncation but may limit the number of characters retrieved, while Factiva might not allow truncation within quoted phrases. These differences necessitated careful standardisation and tailoring of search strings in order to retrieve a manageable and relevant dataset across all three databases.These databases provide access to full-text content from worldwide sources, including local and regional newspapers, newswires, radio and television programme transcripts, press release wires, etc (Eureka 2024; Factiva 2024; Nexis Uni 2024). Based on the research objectives, we created three groups of keywords to search for sources on HAIs: human-animal interaction, animal, and influences (Table 1). The keywords were grouped and selected based on thematic relevance to the review’s focus on HAIs. The selection process was informed by common terminology found in both academic and grey literature, as well as established classifications used in related studies on HAIs, One Health, and One Welfare frameworks.

**Table 1.** Groups of keywords used in the review of human-animal interaction literature: Concepts related to human-animal interaction, animal types, and associated influences

Group	Keywords
Group1: Human-animal interaction	Human-Animal Interaction* (HAI*), Human-Animal Relationship* (HAR*), Human-Animal Bond* (HAB*), Animal-Assisted Interventions (AAI*), Animal-Assisted Therapy (AAT), Pet Ownership*, Pet Guardianship*, Pet Guardian*, Companion Animal Guardian*
Group 2: Animal	Companion Animal*, Pet*, Service Animal*, Therapy Animal*, Agricultural Animal*, Laboratory, Zoo, Aquarium, Wildlife
Group 3: Influences	Social, Health, Well-Being, Wellness, Individual*, Famil*, Communit*, Education

Group one includes terms that explicitly refer to established concepts in the field, such as Human-Animal Interaction (HAI), Human-Animal Bond (HAB), and Animal-Assisted Interventions (AAI). The use of asterisks (e.g. Interaction, Relationship, Bond) allows for retrieval of plural or variant forms (e.g. ‘interactions’, ‘relationships’). Not all abbreviations require an asterisk, as terms like AAT or AAI are already fully formed acronyms. Additional terms such as Pet Ownership and Companion Animal Guardian were included to capture a broader range of informal or lay terms used in grey literature to describe human-animal relationships. Group two encompasses a range of animals involved in different types of HAIs, from pets and service animals to those in institutional or wild settings (e.g. zoos, laboratories, wildlife). Asterisks are used when it is important to capture variations (e.g. Pet includes ‘pets’, ‘petting’; Companion Animal includes both singular and plural). In contrast, terms such as Zoo, Aquarium, and Laboratory are included without asterisks as they are typically used in their singular form and are conceptually specific.

Group three captures key domains of influence or impact associated with HAIs, such as social, health, and educational outcomes. The asterisk is applied to root forms (e.g. Individua, Famil, Communit) to capture diverse suffixes such as ‘individuals’, ‘individualized’, ‘family’, ‘familial’, ‘community’, or ‘communities’. This approach increases the sensitivity of the search, allowing for broader retrieval of relevant literature without limiting it to a single grammatical form.

The search terms were standardised to ensure consistency and comparability across all three databases. Before finalising the grey literature search terms, the team run several rounds of pilot searches across three databases. This standardisation was necessary to identify the most relevant documents while maintaining a manageable dataset. Without a consistent set of terms, the results could have varied significantly across platforms, leading to over-duplication, gaps, or inclusion of irrelevant materials. A structured approach to keyword selection helped streamline the screening process and enhanced the reliability of the dataset.

Since this rapid review explores grey literature’s role in public education on human-animal well-being and welfare, the three groups reflect different types of animals, forms of HAIs, and the health impacts of HAIs across various settings. The Boolean operator “OR” was used within each keyword group, and “AND” was used between the groups to refine the search. To further narrow the results, we focused on grey literature published from 2016 to 2022 and written in English. The search identified 1,994 entries, which were uploaded to Covidence, a web-based platform that facilitates the conduct of a comprehensive literature review (Covidence 2024). After manually removing 259 duplicates and an additional 871 duplicates through Covidence (1,130 in total), 864 entries were moved forward for screening.

### Step 2: Data screening

We developed a set of inclusion and exclusion criteria to guide the selection of relevant grey literature. The inclusion criteria are: (1) entries containing HAI-specific knowledge, strategies, and outcomes addressing diverse health and social issues at the individual, family, and community levels; (2) entries with institutional or organisational backing (e.g. news reports, government sources, organisational newsletters, and non-peer-reviewed journals); and (3) entries potentially contributing to knowledge mobilisation. The inclusion criteria did not require documents to report scientific findings. We excluded personal blogs and social media posts, as well as promotional or commercial materials primarily focusing on selling products, due to concerns about content quality. These criteria also informed the selection of keywords, ensuring alignment with the scope of documents deemed relevant and appropriate for this review (see Table 1).

**Figure 1.**
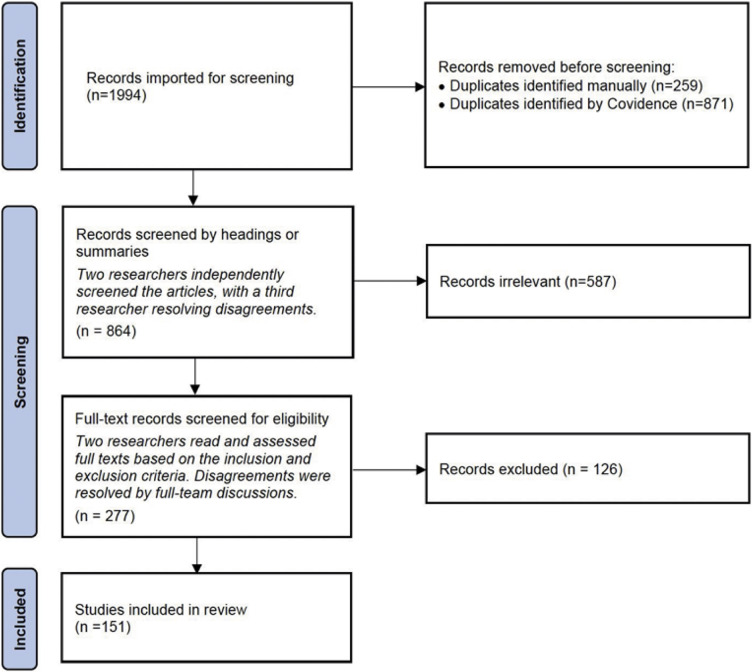
Flowchart illustrating the identification, screening, and selection process of grey literature sources on Human-Animal Interactions (HAI) published from 2016 to 2022 and written in English.

Two team members independently screened the documents by the headings or summaries, with a third researcher resolving any disagreements. This initial screening excluded 587 articles, leaving 277 for the full-text screening. The two researchers then carefully read and assessed the grey literature based on the inclusion/exclusion criteria, with any disagreements being resolved through full-team discussions. This process led to the exclusion of 126 sources, resulting in a final total of 151 entries of grey literature selected for data analysis (Figure 1). The research team has uploaded the list of the included articles to a public research database. Please see the *Data availability statement* (Ru *et al.* 2024) for further details on how to access the data.

### Step 3: Data analysis

We utilised a mixed-methods approach for data analysis. We conducted a univariate analysis to capture basic information from the included grey literature, such as the types of animals, vulnerable populations, and community settings discussed. This was followed by a Reflexive Thematic Analysis (RTA) as outlined by Braun and Clarke (2006, 2019, 2020, 2022), a method grounded in a qualitative paradigm that emphasises the active role of the researcher in identifying and interpreting patterns of meaning across the data. Unlike coding reliability or codebook approaches to thematic analysis, RTA acknowledges researcher subjectivity as an analytic resource rather than a bias to be minimised. It allows for a high degree of flexibility in how themes are generated, which distinguishes this approach from other forms of thematic analysis that rely more heavily on pre-determined coding frameworks or inter-coder reliability.

Using an inductive approach (Azungah 2018), two researchers independently reviewed and coded the grey literature using Excel® spreadsheets. They then met to compare the codes. The involvement of two researchers in the data analysis process allowed for diverse perspectives and interpretations, with any differences resolved through discussions. Ultimately, the research team reviewed the codes and formulated overarching themes and subthemes (Table 2). For example, “*The dog’s presence is calming and comforting for many clients*” (Thompson 2018; paragraph 7) was coded as ‘Mental health’, and “*Pet owners that have participated in such experiments have been found to have lower rates of high blood pressure and lower levels of cortisol*” (Budget Savvy Diva 2022; paragraph 8) was coded as ‘Physical health’. The two codes were then grouped as ‘Individual health and well-being’ under the theme one: *HAIs benefit individual well-being, families, and animal well-being.* We identified three primary themes and eight sub-themes to understand the grey literature. The following sections present the results of the analysis.

**Table 2.** Thematic framework of human-animal interaction (HAI) outcomes organised in themes, subthemes, and associated codes across three overarching domains

Themes	Subthemes	Codes	Key Terms
HAIs benefit individual well-being, families, and animal well-being	Individual well-being	Physical health, mental health, social benefits	Physical activity, cardiovascular health, emotional support, comfort, companionship, isolation
	Positive influences on families	Family	Family ties, family bonds
	Animal well-being	Animal welfare, One Health	Interconnected, pets’ well-being, maltreatment
HAIs promote prosocial behaviours and community development	Prosocial behaviours and community integration	Prosocial behaviour, community ties	Trust, interact with neighbours, community activities, social bonding
	Neighbourhood planning and community built-environment	Community physical environment	Green spaces, animal-friendly places, healthy environment
	Community education	Community education, educational activities	Awareness campaign, seminar, educational program
HAIs and disaster preparedness/response	Resilience capacities	Coping capacity in challenging times	Recover from or cope with crisis, support, comfort, hope
	Including animals in emergency preparedness and response	Disaster preparedness, extreme event responses	Pet support services, rescue, care, shelter

Relevant content was extracted based on both explicit conclusions made by the original authors and implicit themes identified by the reviewers during detailed reading. The extraction focused on sections where authors summarised their findings, discussed implications, or made evaluative claims relevant to HAI. However, the entire text was not coded line-by-line; rather, thematic analysis was applied selectively to content sections most relevant to our research questions.

## Results

The following sections demonstrate the results from both the univariate and thematic analyses, providing an overview of how the grey literature addresses HAIs and their varied impacts.

### Characteristics of the included grey literature

The univariate analysis helps to examine the key characteristics of the articles reviewed, focusing on the locations of HAIs mentioned by the sources, animal species covered, the vulnerable and marginalised populations, and the community settings in which HAIs take place. These aspects provide insights into the scope and diversity of HAIs covered in the grey literature.

#### Geographical regions

**Figure 2.**
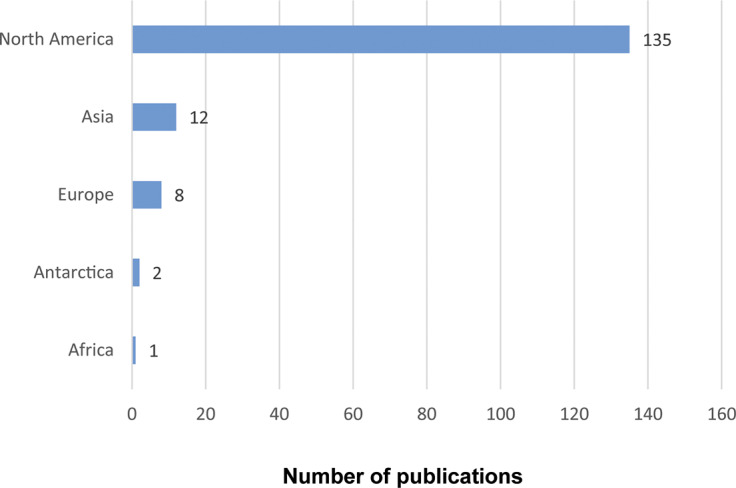
Number of sources covering geographical regions in the grey literature on Human-Animal Interactions (HAI) published from 2016 to 2022 and written in English.

The grey literature spans multiple geographic locations (Figure 2).

The majority of the selected articles focus on HAIs in North America. Twelve sources discuss HAIs in Asia, eight in Europe, two in Antarctica, and one in Africa. Of the articles selected, none address Oceania or South America.

#### Animal species

**Figure 3.**
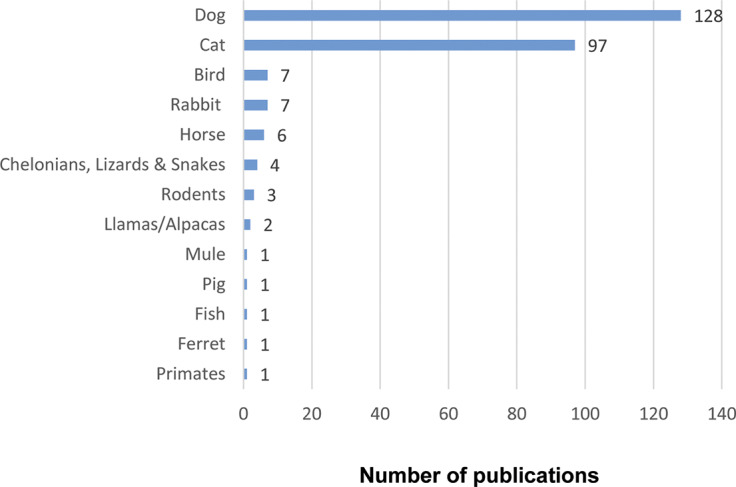
Number of sources covering animal species in the grey literature on Human-Animal Interactions (HAI) published from 2016 to 2022 and written in English.

The grey literature explored a variety of animal species (Figure 3), highlighting the interactions between humans and these different animals. Dogs were the most frequently mentioned, followed by cats. This distribution aligns with trends in academic research, where dogs and cats dominate discussions of HAIs (Thomson *et al.* 2018). Animal species include birds, horses, mules, pigs, fish, rabbits, ferrets, llamas/alpacas, rodents, primates, as well as chelonians, lizards, and snakes.

#### Vulnerable and marginalised populations

**Figure 4.**
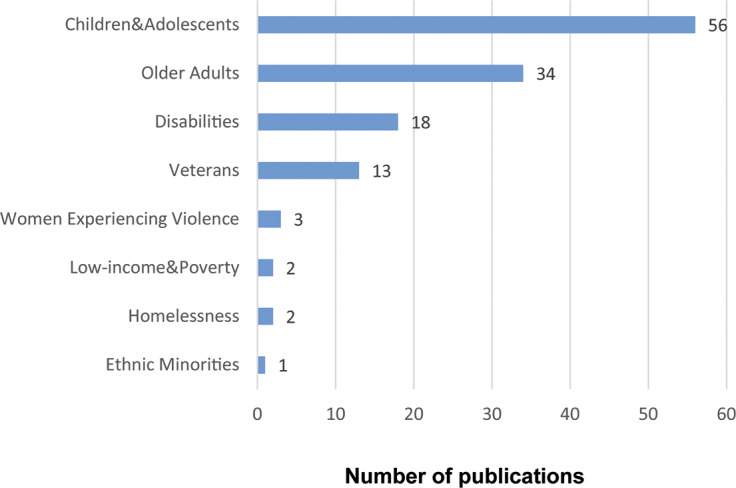
Number of sources covering vulnerable and marginalised populations in the grey literature on Human-Animal Interactions (HAI) published from 2016 to 2022 and written in English.

The grey literature also focused on the interactions between animals and various vulnerable and marginalised groups (Figure 4). The most frequently mentioned vulnerable groups were children/adolescents and older adults. Other groups include women experiencing domestic violence, people with disabilities, veterans, people experiencing financial hardships, people experiencing homelessness, and ethnic minorities. Sources addressing multiple vulnerable populations (e.g. disabled children) were counted in all relevant categories, allowing us to capture the full range of populations represented. Similarly, for species categorisation, papers referring to more than one species were also included in each applicable category.

#### Community settings

**Figure 5.**
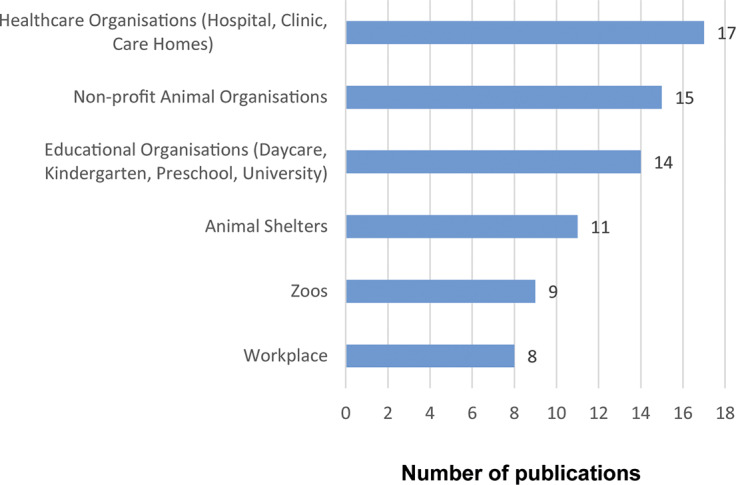
Number of sources covering different community settings in the grey literature on Human-Animal Interactions (HAI) published from 2016 to 2022 and written in English.

HAIs were discussed in various community settings, including zoos, educational organisations (e.g. daycare, kindergarten, pre-school, and universities), healthcare organisations (e.g. hospitals, clinics, care homes), non-profit animal organisations, shelters, and workplace (Figure 5).

### Themes covered by the grey literature

#### Theme 1: HAIs benefit individual well-being, families, and animal well-being

The thematic analysis revealed that the grey literature informed the public about the multifaceted benefits of HAIs, extending beyond individual well-being to positively impact families and the animals themselves. Approximately 67% of the sources address the theme of HAIs (at least partially) in relation to individual physical, emotional, and social well-being, with some highlighting potential contributions to family bonds and improvements in animal welfare. The findings highlight the interconnectedness of human and animal well-being.

##### Sub-theme 1: HAIs benefit well-being of individuals

The vast majority of sources cover the positive influences of HAIs on human individuals’ physical health, mental health, and general well-being (Thompson 2018; Salmon 2020; Budget Savvy Diva 2022), which is also a research focus in scientific literature (Hosey & Melfi 2014; Fine 2019). In this article, well-being is used in the broadest sense and includes physical, mental, and social aspects of health (Carr *et al.* 2018). The grey literature referred to previous research findings to support the health benefits. For example, one article from the PR Newswire shared research results from the Human Animal Bond Research Institute showing that 80% of companion animal guardians reported a reduced sense of loneliness (French 2019). The documents also introduce the ‘feel-good hormones’ triggered by interacting with animals, including dopamine, oxytocin, serotonin, and endorphin, allowing the public to understand the scientific explanations about HAIs’ health benefits (Herzog 2021; Budget Savvy Diva 2022).

Sharing research findings strengthens the recognition of animals as a source of emotional and social support.

The grey literature informs the public that the benefits of HAIs are particularly valuable for vulnerable and marginalised groups, who often have small social networks and limited support from other people. For example, HAIs enhance children’s health (McNeill 2018), promote healthy ageing (Human Animal Bond Research Institute [HABRI] 2020), support veterans experiencing PTSD (US Department of Veterans Affairs 2019), and assist people with chronic diseases or disabilities (Hemingway 2022). This view of animals as a source of support compensating for human support has been demonstrated by empirical studies (Carr *et al.* 2018; Gibson *et al.* 2022). The public also learn about animal-assisted therapy and interventions through grey literature coverage (Parrot Website 2016; Thompson 2018), and the activities “*can assist in the treatment of a broad range of conditions from post-traumatic stress to Alzheimer’s disease to autism spectrum disorder*” (HABRI 2020; paragraph 4).

Notably, some of the articles emphasise that the health benefits are led by positive and friendly interactions with animals (Tarrab 2021). Animals’ behavioural problems can significantly impact the physical health, mental health, and general well-being of individuals (Brulliard 2017).

##### Sub-theme 2: HAIs have positive influences on families

Companion animals are increasingly viewed as family members and integral participants in family life (Carver 2021). The sources suggest that companion animals encourage more family activities, such as family walks (HABRI 2022), which not only promote physical health but also increase the time family members spend together (Banfield Pet Hospital 2021).

The shared family time strengthens relationships and bonds within the family. This is especially beneficial for individuals from a dysfunctional family, as the family members can bond over a companion animal (Kabilan 2022). Additionally, the grey literature highlights how companion animals can engage children in family activities and caregiving responsibilities, which may help foster empathy, responsibility, and stronger family relationships (Baxter 2021).

These examples illustrate that grey literature disseminates knowledge about HAIs’ positive influences in family settings. Empirical research supports these positive impacts by demonstrating that companion animals enhance bonds between family members (Faver & Cavazos 2008). By highlighting these benefits, grey literature offers broader implications for family well-being and public health.

##### Sub-theme 3: HAIs benefit animal welfare

The grey literature emphasises particularly the positive side of HAIs rather than other types of HAI, thus having primarily “*positive effects on both animals and people*” (Tarrab 2021). The One Health framework, promoting the interconnectedness between human, animal, and environmental health, has been introduced to the public (Association of Zoos and Aquariums 2020). Research findings have been shared to indicate that the health benefits of HAIs motivate companion animal guardians (CAGs) to take better care of their companion animals (Baxter 2020), which can foster positive HAIs and benefit the animals’ well-being.

The documents encourage CAGs to continuously learn about their companion animals and how to improve their care (American Humane 2021). For example, the American Humane (2021) suggested that “[p]*ets have emotions just like humans, and to keep pets healthy both physically and mentally, pet owners must feed both the mind and body of their pets*”. CAGs need to ensure their companion animals have “*proper exercise and stimulation*” to improve the animals’ quality of life (American Humane 2021).

The grey literature addresses the welfare of service/therapy animals.

Therapy dog handlers are advised to pay attention to environmental stimuli, closely monitor the animals’ behaviours, and ensure the animals rest as needed during animal-assisted therapy sessions (Thompson 2018). The sources also stress the need for consistent, standardised training based on positive reinforcement — a practice advocated by peer-reviewed literature (Hiby *et al.* 2004) — along with a comprehensive certification process, to enhance the well-being of both service animals and the people they assist (US Department of Veterans Affairs 2019).

Through knowledge mobilisation, grey literature enhances public education on the interdependence between humans and animals, which has the potential to promote animal welfare.

#### Theme 2: HAIs promote prosocial behaviours and community development

Approximately 64% of the sources address HAIs’ impacts at the community level. The social benefits of HAIs not only enhance individual overall well-being but also promote community integration. The demand for animal-friendly facilities influences community planning and built environment, leading to more inclusive public spaces that consider both human and animal well-being. Furthermore, HAIs foster community education through organisations and community events, which help raise awareness and knowledge about the benefits of HAIs and improved animal welfare.

##### Sub-theme 1: Prosocial behaviours enhance community integration

Prosocial behaviours encompass a range of positive behaviours, including friendly interactions, sharing, offering help, and behaviours that reduce stereotypes (Mares & Woodard 2005). The grey literature shares that animals serve as a conversation starter and relationship facilitator, which breaks the ice between strangers and increases social interactions between neighbours (French 2019). Research and survey results are used to show that companion animals improve people’s ability to relate to others and feel empathy for their problems, and CAGs are willing to engage in conversations with someone holding different political views if they know that person is also a CAG (Banfield Pet Hospital 2020).

Additionally, CAGs are more involved in community activities where community members can connect (Carver 2019).

The articles also suggest that HAIs promote healthy social activities within virtual communities. Sharing animal photographs, videos, and stories in online groups and on social media encourages positive social engagement (Casey 2022). These examples reflect that prosocial behaviours generated by HAIs strengthen a sense of community and promote community integration, which aligns with empirical findings (Wood *et al.* 2005; Bulsara *et al.* 2007).

##### Sub-theme 2: HAIs improve neighbourhood planning and community built environment

The frequent HAIs have led to an emphasis on animal-friendly infrastructures and facilities that enhance neighbourhood planning and the built environment. Grey literature demonstrates how animal-friendly designs can be incorporated into different settings.

The growing demand for animal-friendly housing, workplaces, and parks reflects a shift toward more inclusive urban planning that accommodates the needs of both humans and their companion animals (Juric 2019; Serlin 2021). Therapy and service animals are increasingly integrated into hospitals, veterans’ facilities, schools, senior homes, prisons, and rehabilitation services, where they play a critical role in supporting people in need (Juric 2019; Pet Partners 2022). Furthermore, pet-friendly domestic violence shelters are needed to provide a safe place for victims of domestic violence and their companion animals (HABRI 2021). The presence of animals in these settings highlights the importance of improving animal-friendly facilities to reduce barriers to positive HAIs.

The documents also identify changes in zoo environments and management, with special attention to zoo animal physiology and behaviour. The changes include housing zoo animals in enclosures that mimic their natural habitats, better treatment of zoo animals, veterinary care for sick or injured animals, and nutritious diets. These efforts reflect a commitment to prioritising animal welfare and contributing to public education on animals and HAIs (Technology Times 2018).

These examples illustrate how HAIs can promote more inclusive neighbourhood planning and community built environments for both humans and animals. As shown in existing research, animal-friendly infrastructures contribute to healthier, more connected communities (Middle 2020).

##### Sub-theme 3: HAIs generate community education

Sources from zoos, aquaria, non-profit animal organisations, and shelters play a key role in community education about the benefits of HAIs and animal welfare. Through these sources, the public learns about community events organised by these agencies. Educational programmes from zoos and aquaria help people understand animal behaviours, foster empathy for animals, and promote pro-environmental actions to conserve wildlife (Cheyenne Mountain Zoo 2020; Stuart *et al.* 2021). Similarly, sources and events hosted by non-profit animal organisations and shelters educate the public on the benefits of HAIs, encourage adoptions, and raise awareness of pet health (French 2019; American Humane 2021). These efforts encourage people to reflect on the treatment of animals and the relationships with them.

#### Theme 3: HAIs and disaster preparedness/response

About 26 percent of the sources discuss HAIs in disaster and emergency settings, such as COVID-19 and climate-change-induced disasters. The grey literature informs the public about the role of HAIs in building individual and community resilience capacities, as well as the necessity of developing animal-inclusive disaster preparedness and response plans.

##### Sub-theme 1: HAIs support building resilience capacities

One key topic of the grey literature published from 2020 to 2022 is the role of animals in building resilience during COVID-19. Consistent with empirical studies (Kogan *et al.* 2021; Brooks & Greenberg 2023), the included sources show that the health benefits and emotional support provided by companion animals helped people cope with the challenges of the pandemic (Budget Savvy Diva 2022). The companionship and unconditional love offered by companion animals alleviated loneliness and social isolation brought on by social distancing, quarantine, and lockdown measures. Survey results shared by the articles show that companion animals helped companion animal guardians (CAGs) cope with uncertainties caused by the pandemic, reduced their stress, anxiety, and depression, and increased household happiness during the pandemic (Banfield Pet Hospital 2021). The benefits were particularly important for vulnerable populations during COVID-19, such as children (Mars Petcare 2021) and older adults (Hoyle 2020). The positive impact of HAIs in COVID-19 was evidenced by the surge in pet adoption and the number of households with companion animals during this period (Dibdin 2021).

The sources also reminded the public of the unintended effects of co-dependency and separation anxiety associated with the reopening after the pandemic (Camp Bow Wow 2019). These concerns require CAGs to learn more about the causes, symptoms, and behaviours associated with separation anxiety (Camp Bow Wow 2019), which will also prepare CAGs to better handle future emergencies with greater resilience (Dibdin 2021).

The grey literature demonstrates that HAIs play a crucial role in supporting individuals and households during difficult times, contributing to enhanced resilience capacities in disaster and emergency settings.

##### Sub-theme 2: Including animals in emergency preparedness and response

The sources discuss the importance of HAIs during crises, including climate-change-induced disasters, conflicts, and wars (Hurst 2020; Humane Society International 2022). The emphasis on the necessity of animal-inclusive emergency preparedness and response plans (Monacelli & Dado 2019) is also increasingly reflected in peer-reviewed articles (Thompson 2013; Wu *et al.* 2023b; Ru *et al.* 2025). The grey literature encourages companion animal guardians to create an emergency preparedness kit for their companion animals, ensuring that all family members, including animals, are ready when facing emergencies or disasters (Monacelli & Dado 2019).

The importance of animal-inclusive emergency preparedness and response plans is illustrated by the tragic events of Hurricane Katrina, where many people refused to evacuate without their pets, resulting in the loss of human and animal lives (Carver 2019). This situation led to the passing of the PETS Act (Pets Evacuation and Transportation Standards Act), “*which authorizes FEMA* [Federal Emergency Management Agency] *to provide rescue, care, shelter and essential needs for individuals with animals and for animals themselves*” (Hurst 2020).

Support from communities, authorities, and animal organisations is crucial in protecting animal welfare in disasters and emergencies (Carver 2019; Humane Society International 2022). Including animals in emergency preparedness and response efforts promotes the well-being of both humans and animals (Carver 2019).

## Discussion

This rapid review examined 151 grey literature documents focusing on HAIs. The univariate analysis reveals broad coverage, encompassing HAIs with a wide range of animal species, including dogs, cats, rabbits, birds, and more, as well as different types of animals, such as companion animals, therapy animals, and zoo animals. HAIs happen in different settings, from households and hospitals to senior homes, animal organisations, and zoos, and have been acknowledged in different countries and regions. The thematic analysis shows that the sources predominantly discuss the positive health effects of HAIs at the individual, family, and community levels, using anecdotal stories and scientific research findings presented in plain language to reach a broader audience. Current literature underscores the highly context-dependent nature of human-animal interactions (HAI), with outcomes varying significantly based on several interrelated factors. Research shows that the quality and depth of the human-animal bond play a critical role in shaping both human and animal well-being, with stronger attachments often associated with more positive outcomes (Gee *et al.* 2016. However, these benefits are not universal; individual animal temperament, species-specific behaviours, and prior training also influence the interaction’s impact, particularly in therapeutic or support settings (Beetz *et al.* 2012). Moreover, the goals and environmental context, whether in a clinical, educational, domestic, or disaster response setting, can moderate the effectiveness and ethical implications of HAIs (Wu *et al.* 2021, 2023b). As such, outcomes cannot be generalised across all HAI scenarios, reinforcing the need for nuanced, evidence-based approaches that consider the specific relational, situational, and gender-specific variables at play (Wu *et al.* 2023c).

A closer review of the source shows that most stories, research, and practices regarding HAIs are in Western countries, such as the United States and Canada. There is a need for further investigation and practical application in ‘non-Western’ and non-English speaking countries. The use of English as an inclusion criterion likely contributed to the overrepresentation of documents from English-speaking countries, particularly the US and Canada. Future research can also explore the relationship between cultural background and people’s perceptions of animals and HAIs, and how these perceptions impact human-animal welfare in different cultural contexts.

A better understanding of HAIs and their health impacts can help the public to recognise animals as a valuable source of emotional and social support, providing an additional coping strategy during stressful events (Carr *et al.* 2018; Wu *et al.* 2023b). Service providers can better assist vulnerable and marginalised populations by incorporating HAIs into therapeutic strategies and support systems, as these groups often have limited access to human support (Muraco *et al.* 2018).

The grey literature illustrates the effectiveness of HAIs in practices, as evidenced by animal-assisted therapy and animal-assisted interventions, as well as the use of service animals for people with disabilities and veterans experiencing PTSD. However, previous research has revealed a lack of public knowledge regarding service animals, as service dog users often face unwanted attention or even discrimination in public places (Sanders 2000; Gibson *et al.* 2022). Future grey literature can contribute to public education by clarifying the differences between companion animals, therapy animals, and service animals, as well as specific types of service animals, such as guide dogs, psychiatric service dogs, and more (Nova Scotia undated).

Enhancing public awareness can help address challenges faced by service animals and their users and maximise the benefits of HAIs. As the family structure changes and family size decreases, multispecies families, which refer to households that include companion animals as integral family members, have become more common (Irvine & Cilia 2017). The grey literature emphasises the crucial role of companion animals in strengthening family ties by providing emotional support and fostering a sense of belonging within households (Fine 2019). At the community level, the social benefits of HAIs encourage interactions among neighbours, contributing to healthier neighbourhood relations, greater community connectedness, and enhanced community integration. Service providers can apply the benefits of HAIs at the family and community levels to support families in need and create more inclusive and supportive community environments, which can in turn improve both human and animal welfare.

The analysed articles demonstrate that positive HAIs are reciprocal and mutually beneficial, contributing to public education on One Health and raising public awareness about protecting animal welfare. The grey literature suggests that animals can benefit directly from interacting with humans, as the health effects of certain types of HAIs are also observed in animals (Gee *et al.* 2016). However, although some research findings have shown that companion animals interacting with their guardians can lower animals’ heart rates and stress levels (Beetz *et al.* 2012; Edwards *et al.* 2022), research exploring HAIs’ health effects on animals remains limited. Future interdisciplinary research can further examine these reciprocal relationships and explore how grey literature reflects or reports on the mutual benefits of HAIs.

In addition to the health benefits of direct HAIs, animals also benefit from better treatment. The grey literature promotes a deeper understanding of animals’ essential roles in human life and society, which in turn encourages the public to reconsider their treatment of animals. The sources advocate for responsible companion animal guardianship, which addresses the physical and mental health of companion animals, prevents animal behaviour problems, reduces the likelihood of relinquishment and abandonment, and minimises conflicts between companion animal guardians and people living without companion animals (Westgarth *et al.* 2019). The articles also suggest that animal organisations and zoos improve their treatment of animals, particularly in response to growing public awareness, evolving animal welfare standards, and increased scrutiny from advocacy groups. Therapy/service animal trainers, as well as zoo staff, are encouraged to respect animals’ needs and protect their welfare rather than viewing them as tools for providing services or entertainment. These recommendations foster healthy HAIs and contribute to a more ethical and compassionate treatment of animals across various settings.

Reconsidering the treatment of animals is also reflected in the grey literature through its emphasis on animal-inclusive community planning and built environment. Households with companion animals often face challenges in finding affordable, good-quality, and stable animal-friendly rental housing (Power 2017). This issue has become more complex after COVID-19 due to increasing companion animal guardianship and an urban housing crisis that is characterised by rising rents and a shortage of housing supply (Bryden-Blom 2023). Adequate animal-friendly housing, supported by clear legislation and policies protecting housing security of multispecies families, can prevent pet relinquishment and promote human-animal well-being (Applebaum *et al.* 2021). The analysed grey literature calls for the development of animal-friendly facilities within various community settings, such as parks, schools, and hospitals. Future research could explore how universal design, which is the design of buildings and environments to make them accessible to people with a wide range of ages, abilities, and other characteristics (Steinfeld & Jordana 2012), can contribute to inclusive community planning that benefits both humans and animals.

Both humans and animals face increasing risk from more frequent disasters and emergencies (Wu *et al.* 2023b). The documents recognise the impacts of HAIs on both immediate responses and longer-term recovery during crises, emphasising the necessity to incorporate animals into emergency preparedness and response plans. Efforts such as pet emergency kits, animal-friendly shelters for multispecies families, temporary homes and rescue plans for companion animals, farm animals, and wildlife facilitate disaster and emergency management. Further research is needed to explore the needs and challenges faced by people living or working with animals, including companion animal guardians, farm owners, shelter staff, etc, in developing animal-inclusive emergency preparedness and response plans. The findings can help communities, animal organisations, and authorities better support HAIs in disaster and emergency settings. Comprehensive emergency preparedness and response plans that consider HAIs can promote human-animal welfare, prevent loss, and strengthened animal, individual, and community resilience.

There are several research limitations to this grey literature rapid review. The first is related to the credibility and quality of grey literature available online. Although including grey literature can provide a more comprehensive understanding of a topic, the sources are not peer-reviewed, raising concerns about clarity (Mahood *et al.* 2014). To address this concern, two researchers independently screened and selected relevant grey literature, with the third resolving any disagreements. The inclusion criteria did not filter documents based on peer-review status, credibility, or potential bias, though materials were assessed for relevance and clarity by three researchers. We have also elaborated on the role of multiple reviewers as a quality check for consistency in interpretation rather than a formal credibility assessment. This process could help include relevant and qualified sources. Additionally, the rapid review provides an initial examination of current grey literature on HAIs and explores its role in public education about human-animal welfare. The findings related to HAIs partially align with academic debates presented in peer-reviewed journal articles, which discuss both the benefits and potential drawbacks of HAI, “*including such areas as pet death, zoonotic risks, and allergens that may affect human health*” (Wilson & Barker 2003; p 17). Future research should critically examine how grey literature presents research findings and its potential role in spreading an overly positive picture of HAI that could influence public education and policy decisions related to human and animal welfare.

The second limitation considers the scope of grey literature, which is affected by its formats, time-frames, and written languages. This rapid review adapted the PRISMA approach to grey literature review and only focused on newsletters and reports from organisations in English between 2016–2022. This criteria omitted other formats of sources and grey literature published in other languages or outside this time-frame, which could limit the comprehensiveness of the review. Future research can conduct a systematic review using the PRISMA approach to further examine the emerging knowledge about HAIs.

### Animal welfare implications

The evidence of HAIs’ positive effects on individuals, families, and communities shared by grey literature can empower individuals, families, and service providers to incorporate animal support into everyday life and services. Moreover, the analysed articles emphasise the reciprocal nature of HAIs, challenging traditional human-animal boundaries. The essential role of animals in human lives and society calls for strategies that promote animal welfare through the development of animal-friendly facilities, inclusive community planning, and disaster management policies that explicitly account for animals. The thematic analysis conducted in this review demonstrates that when animal needs are systematically integrated into broader human systems, such as public health, emergency response, housing, and social support, both human and animal welfare are significantly improved. This interconnectedness reinforces the rationale for animal-inclusive policies as not only ethically sound but also socially beneficial.

Importantly, the synthesis of grey literature reveals a strong foundation of community-based insights and institutional initiatives that bridge the gap between academic research and real-world application. These non-academic sources provide accessible, practical knowledge that informs public discourse and can drive change at grassroots and policy levels. By bringing together narratives, case studies, and organisational practices, the review highlights how attention to animal welfare, particularly in disaster contexts and vulnerable communities, can strengthen social cohesion, improve emergency outcomes, and enhance emotional and psychological well-being.

The findings of this review are particularly relevant for policy-makers, urban and disaster planners, public health officials, animal welfare organisations, and community-based practitioners. These audiences are in positions to implement cross-sectoral policies that accommodate the presence and needs of animals in various public domains. Moreover, the review encourages researchers and decision-makers to collaborate in producing accessible outputs that resonate with a wider audience beyond academia.

To extend the impact of this work, we propose developing a companion piece of grey literature, such as a policy brief, infographic summary, or community guide, that translates the thematic analysis into clear, actionable recommendations. This supplementary material would be tailored for diverse stakeholders, including community leaders, animal guardians, NGOs, and municipal planners, aiming to enhance understanding of the mutual benefits of human-animal interactions and provide concrete steps for implementing animal-inclusive policies in everyday practice.

In doing so, the review not only advances scholarly discourse but also contributes to practical solutions that uphold and improve animal welfare while fostering more inclusive, resilient, and compassionate communities.

## Conclusion

This rapid review provides a synthesis of grey literature in English from three databases (Eureka, Factiva, and NexisUni) between 2016 and 2022. The analysis shows the diversity and wide scope of HAIs discussed in the sources, which inform the public about the essential role of animals in human society. By translating theoretical concepts and research findings into accessible information, grey literature serves as a valuable tool for knowledge mobilisation, bridging the gap between researchers, service providers, decision-makers, and the general public (Babarczy *et al.* 2024).

## Data Availability

**Ru S, Hernandez DV and Wu H** 2024 Human-animal interactions: A review of grey literature. *Open Science Framework.* https://doi.org/10.17605/OSF.IO/8VJY7.
